# Predictive diagnostic value of the tourniquet test for the diagnosis of dengue infection in adults

**DOI:** 10.1111/j.1365-3156.2010.02641.x

**Published:** 2011-01

**Authors:** Mayfong Mayxay, Rattanaphone Phetsouvanh, Catrin E Moore, Vilada Chansamouth, Manivanh Vongsouvath, Syho Sisouphone, Pankham Vongphachanh, Thaksinaporn Thaojaikong, Soulignasack Thongpaseuth, Simmaly Phongmany, Valy Keolouangkhot, Michel Strobel, Paul N Newton

**Affiliations:** 1 Wellcome Trust – Mahosot Hospital – Oxford Tropical Medicine Research Collaboration, Mahosot HospitalVientiane, Lao PDR; 2 Faculty of Postgraduate Studies, University of Health SciencesVientiane, Lao PDR; 3 Centre for Clinical Vaccinology and Tropical Medicine, Churchill Hospital, University of OxfordOxford, UK; 4 Institut de la Francophonie pour la Médecine TropicaleVientiane, Lao PDR

**Keywords:** dengue, tourniquet test, diagnosis, adult, Laos

## Abstract

**Objective:**

To examine the accuracy of the admission tourniquet test in the diagnosis of dengue infection among Lao adults.

**Methods:**

Prospective assessment of the predictive diagnostic value of the tourniquet test for the diagnosis of dengue infection, as defined by IgM, IgG and NS1 ELISAs (Panbio Ltd, Australia), among Lao adult inpatients with clinically suspected dengue infection.

**Results:**

Of 234 patients with clinically suspected dengue infection on admission, 73% were serologically confirmed to have dengue, while 64 patients with negative dengue serology were diagnosed as having scrub typhus (39%), murine typhus (11%), undetermined typhus (12%), Japanese encephalitis virus (5%), undetermined flavivirus (5%) and typhoid fever (3%); 25% had no identifiable aetiology. The tourniquet test was positive in 29.1% (95% CI = 23.2–34.9%) of all patients and in 34.1% (95% CI = 27.0–41.2%) of dengue-seropositive patients, in 32.7% (95% CI = 23.5–41.8) of those with dengue fever and in 36.4% (95% CI = 24.7–48.0) of those with dengue haemorrhagic fever. Interobserver agreement for the tourniquet test was 90.2% (95% CI = 86.4–94.0) (Kappa = 0.76). Using ELISAs as the diagnostic gold standard, the sensitivity of the tourniquet test was 33.5–34%; its specificity was 84–91%. The positive and negative predictive values were 85–90% and 32.5–34%, respectively.

**Conclusions:**

The admission tourniquet test has low sensitivity and adds relatively little value to the diagnosis of dengue among Lao adult inpatients with suspected dengue. Although a positive tourniquet test suggests dengue and that treatment of alternative diagnoses may not be needed, a negative test result does not exclude dengue.

## Introduction

Dengue infection is an increasing public health problem in tropical and subtropical countries, with an estimated 36 million symptomatic cases globally each year and 2.1 million severe dengue cases (Anonymous 2010). In the Lao PDR (Laos), dengue is an important cause of morbidity and mortality in children and adults; epidemics occur every few years (Peyerl-Hoffmann *et al.* 2004; Blacksell *et al.* 2007). In 2009, 14 439 cases of dengue were reported in Laos, with 12 deaths (NCLE 2009).

Dengue infection has conventionally been classified into three grades of severity – dengue fever (DF), dengue haemorrhagic fever (DHF) and dengue shock syndrome (DSS) (WHO 1997) or more recently as dengue, dengue with warning signs and severe dengue (WHO 2009). DF is characterized by sudden onset of high-grade fever with non-specific symptoms, and most cases resolve without specific treatment. DHF is caused by increased vascular permeability and may progress to hypovolaemic shock and potentially lethal DSS (WHO 1997). The laboratory diagnosis of dengue is usually based on serological ELISA tests, which detect specific IgM or IgG antibodies and/or NS1 antigen during the acute phase of infection or a fourfold rise in antibody titre in paired sera. However, these methods are not commonly available in many dengue-endemic countries. Although clinical diagnosis of DSS is usually straightforward, DF and DHF can be difficult to distinguish clinically from a wide range of undifferentiated fevers, such as typhoid, typhus, malaria, leptospirosis and chikungunya virus infection.

Commercial rapid diagnostic tests for the diagnosis of dengue infection are available, but many are neither as sensitive nor specific as their manufacturer’s claim (Blacksell *et al.* 2007). In Laos, diagnosis of dengue is usually based on clinical symptoms and signs because of unavailability of laboratory diagnostic techniques. The tourniquet test (WHO 1997) is widely used among Lao clinicians for the diagnosis of dengue and is simple, needing only a template (below), a sphygmomanometer, stethoscope and watch. It reflects both capillary fragility and thrombocytopenia and was recommended for use in the diagnosis of DHF and DSS but not for DF (WHO 1997). However, in the revised WHO (2009) guidelines, the tourniquet test is listed as a diagnostic criterion for dengue, dengue with warning signs and severe dengue. However, an evaluation of the tourniquet test in the diagnosis of dengue infection among Vietnamese children suggested that it added little diagnostic utility in hospitalized children with low sensitivity (42%) but high specificity (94%) (Phuong *et al.* 2002). In contrast, among Malaysian children, the tourniquet test had fairly good sensitivity (83%) but very low specificity (23.5%) (Norlijah *et al.* 2006). The predictive diagnostic value of the tourniquet test has not been evaluated among adults with suspected dengue with serological confirmation (Wali *et al.* 1999). Therefore, we performed a prospective study to compare the sensitivity and specificity of the admission tourniquet test *vs.* serologically confirmed dengue in adult Lao inpatients.

## Methods

The study was conducted between October 2006 and October 2007 at the Adult Infectious Disease Ward of Mahosot Hospital, a 400-bed primary to tertiary-care hospital in Vientiane Capital (Phongmany *et al.* 2006). The study was approved by the Lao National Ethics Committee for Health Research of Laos and the Oxford Tropical Research Ethics Committee, UK.

### Patients and clinical procedures

Adult patients (aged >15 years) admitted with undifferentiated fever of <7 days with a clinical diagnosis of dengue infection by the admitting physicians were enrolled in the study provided they gave informed written consent. Suspected dengue was not defined as we wished to evaluate the tourniquet test in a real-life situation. All doctors were aware of the WHO case definition (World Health Organisation 1997). On the day of admission, one of the investigators (MM) took the medical history, performed a physical examination and recorded the clinical details on a standard questionnaire. The dengue tourniquet test was then performed according to the standardized method (WHO 1997) within 24 h of admission by one study team member (MM) while the patient lay on a bed. A standard 2.5 cm^2^ square aluminium window, made specifically for the study, was placed on the anterior (volar) aspect of forearm distal to the elbow crease and a line drawn on the skin at the edges of the window using a black ink marker. The mercury sphygmomanometer cuff was inflated, to the mean of systolic and diastolic pressures for a timed 5 min, on the contralateral side to venepuncture. The observer remained with the patient and, at 5 min, removed the cuff and counted the total number of petechiae visible inside the square. Two Lao physicians (SS and PV) of similar clinical experience who had been using the tourniquet test for 18 years each were retrained in the WHO (1997) technique by MM. The second pre-designated observer, depending on who was present on the ward, then immediately counted the total number of petechiae inside the same square and recorded the results. The first and second observers did not confer about the number of the petechiae. The tourniquet test was considered positive when 20 or more petechiae were observed in the 2.5 cm^2^ square.

### Laboratory investigation

Admission blood samples were taken for complete blood counts and malaria smear, biochemistry, blood culture (Phetsouvanh *et al.* 2006) and serum stored at −30 °C for subsequent serology. A convalescent serum was requested 7–14 days after discharge.

Dengue and Japanese encephalitis (JE) infection were diagnosed using PanBio ELISA kits (JE/Dengue IgM Combo ELISA (Cat No. EJED01C), dengue IgG capture ELISA (Cat No. EDEN02G) and Dengue Early ELISA (detects NS1, Cat No. EDEN01P) (Blacksell *et al.* 2008) and read using a Multiskan EX ELISA plate reader (Labsystems, MA, USA) (Moore *et al.* in prep.). All plates were repeated if the positive, negative or calibrator samples were out of range. Kit instructions were followed for the interpretation, and Panbio Units were calculated by multiplying the index value (calculated by dividing the sample absorbance by the cut-off value, which is the average absorbance of the three calibrators) by 10. For the IgM and NS1 ELISA kits, the result was negative if <9, equivocal between 9 and 11 and positive if >11. If both the dengue and JE IgM results were positive, the JE result was divided by the dengue result to give a ratio ≥1 indicating JE infection and <1 indicating dengue infection. The results for the IgG ELISA were recorded as negative if <18, equivocal between 18 and 22 and positive if >22. All equivocal results were repeated. If the repeat result was also equivocal, it was considered negative. We used an algorithm (Table 1, Moore *et al.* in preparation) developed with PanBio Ltd. and Stuart Blacksell to determine each patient’s infection status according to the results from the three ELISA assays.

**Table 1:** Algorithm to determine each patient’s infection status according to the results from JE/Dengue IgM Combo ELISA, dengue IgG capture ELISA and Dengue Early ELISA (detects dengue NS1 antigen)

	Acute serum			Convalescent serum	
		
	IgM	NS1	IgG	IgM	IgG
Dengue					
Primary infection	Positive dengue	Positive or negative	Negative	Positive dengue or negative	Negative
	Negative	Positive or negative	Negative	Positive dengue	Negative
Secondary infection	Positive dengue or negative	Positive or negative	Positive	Positive dengue or negative	Positive or negative
	Negative	Negative	Positive or negative	Positive dengue or negative	Positive
Infection	Negative	Positive	Negative	Negative	Negative
JE					
Positive	Positive JE	Negative	Positive or negative	Positive JE or negative	Positive or negative
	Negative	Negative	Negative	Positive JE	Positive or negative

Scrub and murine typhus were diagnosed using indirect immunofluorescence assays as previously reported (Phetsouvanh *et al.* 2009). A positive result was defined as an IgM or IgG titre ≥1:400 or a fourfold increase in titre (Coleman *et al.* 2002)*.* Two blood cultures (Pharmaceutical Factory No 2, Vientiane) were performed as described previously (Phetsouvanh *et al.* 2006).

### Dengue case definition and management

Each patient’s clinical information and dengue serology were reviewed by two doctors (MM and VC) to assign a consensus final dengue diagnosis using WHO case definitions for DF, DHF and DSS (WHO 1997) and dengue, dengue with warning signs and severe dengue (WHO 2009) independent of the tourniquet test results. Patients were treated according to the hospital treatment guidelines (WHO 1997).

### Statistical analysis

Data were analysed using SPSS v11.0 (SPSS, Chicago, USA). The sensitivity, specificity, positive predictive value and negative predictive value of the dengue tourniquet test in predicting dengue infection at admission were calculated (Altman 1991). Comparisons between two groups were made by the Mann–Whitney *U*, Student’s *t*, chi-square and Fisher’s exact tests, as appropriate.

## Results

A total of 277 patients with clinically suspected dengue infection were admitted, and of these, 234 (84%) were enrolled in the study (Table 2). The reasons for non-recruitment were that the patients were admitted on the weekend or the study team was notified ≥24 h after patients’ admission. The overall mean (95% CI) age (years) and days of fever at presentation of all study patients were 24.2 (23.0–25.3) and 4.6 (4.4–4.8), respectively.

**Table 2:** Admission demographic and clinical details for patients enrolled in a study evaluating the predictive diagnostic accuracy of the tourniquet test for the diagnosis of dengue infection among adult patients in Laos

Variable	All (*n* = 234)	Dengue +ve (*n* = 170)	Dengue −ve (*n* = 64)	*P*-value
Sex, Male, no (%)	127 (54)	91 (54)	36 (56)	0.71
Age (years)	24.2 (23.0–25.3)	22.8 (21.8–23.8)	27.8 (24.5–31.0)	<0.001
Lao Loom ethnicity, no (%)	232 (99)	168 (99)	64 (100)	–
Day of illness (days)	5.1 (4.9–5.4)	5.1 (4.9–5.4)	5.2 (4.7–5.6)	0.84
Day of fever (days)	4.6 (4.4–4.8)	4.7 (4.4–4.9)	4.3 (3.9–4.7)	0.14
Temperature (°C)	38.0 (37.9–38.1)	38.0 (37.9–38.2)	37.9 (37.6–38.2)	0.41
Pulse (beats/min)	85.6 (84.0–87.0)	85.6 (83.8–87.4)	85.4 (82.4–88.3)	0.87
Systolic blood pressure (mmHg)	105.3 (103.5–107.1)	104.4 (102.5–106.3)	107.9 (103.2–112.6)	0.12
Respiratory rate (/min)	21.7 (21.3–22.0)	21.6 (21.2–21.9)	21.8 (20.9–22.8)	0.48
Chills, no. (%)	76/233 (33)	49/170 (29)	27/63 (43)	0.042
Headache, no. (%)	206/234 (88)	149/170 (88)	57/64 (89)	0.76
Joint pain, no. (%)	115/234 (49)	87/170 (51)	28/64 (44)	0.31
Muscle pain, no. (%)	173/234 (74)	127/170 (75)	46/64 (72)	0.66
Retro-orbital pain, no. (%)	151/234 (64.5)	112/170 (66)	39/64 (61)	0.48
Back pain, no. (%)	45/162 (28)	33/111 (30)	12/51 (23.5)	0.41
Nausea, no. (%)	94/233 (40)	74/169 (44)	20/64 (31)	0.08
Vomiting, no. (%)	69/233 (30)	48/169 (28)	21/64 (33)	0.51
Dysuria, no. (%)	10/234 (4)	4/170 (2)	6/64 (9)	0.028
Diarrhoea, no. (%)	42/224 (19)	34/162 (21)	8/62 (13)	0.16
Constipation, no. (%)	7/169 (4)	3/118 (2.5)	4/51 (8)	0.11
Cough, no. (%)	46/232 (20)	32/170 (19)	14/64 (22)	0.60
Difficult breathing, no. (%)	2/232 (1)	1/168 (0.5)	1/64 (1.5)	0.47
Petechia, no. (%)	8/223 (3.5)	7/164 (4)	1/59 (2)	0.68
Gum bleeding, no. (%)	18/223 (8)	18/164 (11)	0	0.004
Nose bleeding, no. (%)	12/223 (5)	12/164 (7)	0	0.03
Vaginal bleeding, no. (%)	5/223 (2)	5/164 (3)	0	0.33
Haematemesis, no. (%)	2/223 (1)	2/164 (1)	0	1.00
Malena, no. (%)	5/223 (2)	5/164 (3)	0	0.33
Any bleeding site, no. (%)	81/234 (35)	74/170 (43.5)	7/64 (11)	<0.001
Rash, no. (%)	96/234 (41)	83/170 (49)	13/64 (20)	<0.001
Abdominal pain, no. (%)	28/234 (12)	19/170 (11)	9/64 (14)	0.54
Sore throat, no. (%)	16/232 (7)	8/168 (5)	8/64 (12.5)	0.038
Haematocrit (%)	42.6 (41.8–43.5)	43.1 (42.2–44.0)	41.3 (39.2–43.3)	0.07
Platelets (cells/mm^3^)	175 335 (169 761–180 908)	171 898 (165 663–178 134)	185 218 (172 964–197 472)	0.04
WBC (cells/mm^3^)	7399 (7028–7769)	6948 (6579–7317)	8759 (7840–9677)	<0.001
Neutrophils (%)	60.3 (58.8–61.8)	58.6 (57.0–60.2)	65.1 (61.9–68.4)	<0.001
Lymphocytes (%)	37.2 (35.7–38.6)	38.8 (37.2–40.4)	31.9 (28.8–35.2)	<0.001

Data are presented as mean values (95% CI), unless otherwise indicated.

### Diagnosis

The classification of patients by the WHO (1997, 2009) dengue grades were not altered when the final tourniquet results, using the results from either observer, were included. Of 234 patients with clinically suspected dengue infection all had both admission and convalescent sera tested and 170 (73%) were serologically confirmed to have dengue. Those with negative dengue serology (*n* = 64) had laboratory evidence for scrub typhus in 25 (39%), murine typhus in 7 (11%), undetermined typhus infection (either scrub or murine typhus) in 8 (12%), Japanese encephalitis virus infection in 3 (5%), undetermined flavivirus infection in 3 (5%) and typhoid in 2 (3%) patients. No laboratory diagnosis was made for 16 (25%) of patients without evidence of dengue infection.

Of those with proven dengue, 63 (37%) had primary and 107 (63%) had secondary infection. Using WHO criteria (1997), 101 (59%) and 66 (39%) of the patients with confirmed dengue were classified as DF and DHF, respectively. Three patients (2%) were unclassifiable because of inadequate information, and no patients with DSS were found. Of all patients with positive dengue serology, 60 (35%) patients were classified as dengue with warning signs; none fulfilled the criteria for severe dengue as described in WHO (2009).

### Tourniquet test

Tourniquet tests, as interpreted by MM, were positive in 68/234 (29.1%; 95% CI = 23.2–34.9%) patients and in 58 (34.1%; 95% CI = 27.0–41.2) of dengue seropositive patients (Table 3). The proportion (95% CI) of patients with positive tourniquet tests was 25.4 (14.6–36.1)% in those with primary and 39.2 (30.0–48.5)% in those with secondary dengue (*P*=0.07). The proportion of patients with positive tourniquet tests was 32.7% (95% CI = 23.5–41.8) and 36.4% (95% CI = 24.7–48.0) among patients with DF and DHF, respectively. The tourniquet test was positive in 41.7% (95% CI 29.2–54.1) of 60 confirmed dengue patients with warning signs and in 30.0% (95% CI 21.4–38.6) of 110 patients with positive dengue serology without warning signs (WHO 2009). Using the ELISAs as a gold standard, the sensitivity and specificity of the tourniquet tests in the diagnosis of dengue (of all grades of severity) were 34% and 84% for the first and 33.5% and 91% for the second observers, respectively. The positive and negative predictive values for the two observers were 85–90% and 32.5–34%, respectively. Of the patients with no serological evidence for dengue infection, 15.6% (10/64) (95% CI 6.7–24.5) had positive tourniquet tests. These patients had diagnoses of undetermined flavivirus infection [3/10 (30%)], scrub typhus [2/10 (20%)], Japanese encephalitis virus [1/10 (10%)] and no laboratory diagnosis [4/10 (40%)]. The tourniquet test was positive in 5% (95% CI 1.7–11.7) of those with typhus and in 8% (95% CI 2.6–18.6) of those with scrub typhus.

**Table 3:** Admission tourniquet test results for 234 patients with suspected dengue by diagnostic categories

Diagnosis	Tourniquet testPositive/total (%, 95% CI)
DF (WHO 1997)	33/101 (32.7, 23.5–41.8)
DHF (WHO 1997)	24/66 (36.4, 24.7–48.0)
Unclassifiable (WHO 1997)	1/3 (33.3, −20–86.7)
Dengue (WHO 2009)	33/110 (30.0, 21.4–38.6)
Dengue with warning signs (WHO 2009)	25/60 (41.7, 29.2–54.1)
Primary dengue	16/63 (25.4, 14.6–36.1)
Secondary dengue	42/107 (39.2, 30.0–48.5)
Not dengue infection	10/64 (15.6, 6.7–24.5)
All typhus	2/40 (5, −1.7–11.7)
Scrub typhus	2/25 (8, −2.6–18.6)
Total	68/234 (29.1, 23.2–34.9)

Dengue fever (DF) was defined as fever with two or more of the following: headache, retro-orbital pain, myalgia, arthralgia, rash, and haemorrhagic manifestations (skin petechiae, bruising, mucosal/gastrointestinal bleeding) and positive serology.

Dengue haemorrhagic fever (DHF) was defined as the above DF symptoms and signs plus spontaneous bleeding (petechiae, ecchymoses or purpura, bleeding from mucosa, gastrointestinal tract, injection sites or other sites, haematemesis or melaena), platelet count <100 000 mm^3^, haemoconcentration, plasma leakage (pleural effusions, ascites or hypoproteinaemia) plus positive serology. Dengue shock syndrome was defined as a combination of criteria of DHF with signs of circulatory failure (rapid and weak pulse, narrow pulse pressure (≤20 mmHg) or hypotension for age (systolic <90 mmHg), cold and clammy skin and restlessness plus positive serology. Patients who did not have all criteria for DHF, but had positive serology, were classified as DF. Patients with negative serology were considered not to have dengue infection.

Dengue without warning signs was defined as fever with two or more of the following: nausea or vomiting, rash, muscle ache and pains, and leukopenia.

Dengue with warning signs was defined as the above dengue without warning signs plus any of the following: abdominal pain or tenderness, persistent vomiting, clinical fluid accumulation, mucosal bleed, lethargy or restlessness, liver enlargement >2 cm and increased haematocrit concurrent with rapid decrease in platelet count.

The percentage (95% CI) agreement in reading the tourniquet tests (as positive or negative) between the first and second observers was 90.2% (86.4–94.0) with Kappa = 0.76, which is classified as good agreement (Landis & Koch 1977). The sensitivity and specificity of the tourniquet test in predicting all typhus were 5% and 66%, respectively and in predicting patients with scrub typhus alone 8% and 68%, respectively.

### Comparison between patients with and without dengue infection

Patients with dengue infection were significantly younger than those without [mean (95% CI) age (years) = 22.8 (21.8–23.8) *vs.* 27.8 (24.5–31.0), *P*<0.001]. The proportions of the patients with admission chills (43%*vs.* 29%, *P*=0.04), dysuria (9%*vs.* 2%, *P*=0.02), and sore throat (12.5%*vs.* 5%, *P*=0.03) were significantly higher in those without dengue compared to those with dengue (Table 2). None of the patients without dengue had gum or nose bleeding, but these were found in 11% and 7%, respectively, of dengue seropositive patients (*P*=0.004 and *P*=0.03, respectively). Patients with dengue infection had a significantly higher frequency of admission rash compared with those without (49%*vs.* 20%, *P*<0.001). Admission mean (95% CI) platelet and white cell counts were significantly lower in patients with dengue than in those without dengue infection [171 898 (165 663–178 134) *vs.* 185 218 (172 964–197 472) mm^3^, *P*=0.04 and 6948 (6579–7317) *vs.* 8759 (7840–9677) mm^3^, *P*<0.001, respectively].

## Discussion

This study suggests that, in Lao hospitalized adults, the conventional tourniquet test adds relatively little predictive diagnostic value for the diagnosis of dengue infection, with low sensitivity but good specificity. This finding is consistent with a similar study in Vietnamese children, which also showed low sensitivity (42%) but good specificity (94%) of the tourniquet test in the diagnosis of dengue (Phuong *et al.* 2002). There is only one published prospective evaluation of the tourniquet test in adults admitted with dengue; the test was positive in only 27% of DHF cases. However, there was no serological confirmation of dengue (Wali *et al.* 1999). Although the tourniquet test was not found to have much clinical utility in a Lao hospital setting, it may, with high specificity, be useful in identifying patients outside of the capital, where laboratory facilities are not available and epidemics are important, provided that the low sensitivity is recognized. Limitations of the study included that the results from two ‘second’ tourniquet test study readers were pooled, no patients with DSS were included in the study and we did not evaluate the usefulness of tourniquet tests after admission.

In the 1997 World Health Organization guidelines for dengue diagnosis, the tourniquet test, is one of the criteria for the diagnosis of DHF but not for DF (WHO 1997). In this study, 33% of patients with serologically confirmed DF had positive tourniquet tests, while 64% of patients with DHF had negative tourniquet tests. Therefore, if the admission tourniquet test is used to diagnose DHF as distinct from DF, clinicians may frequently misclassify DF as DHF and patients with DHF may be misdiagnosed as having DF or not having dengue infection if the tourniquet test is the main discriminator. In the study of Phuong *et al.* (2002), approximately 40% of children with confirmed dengue infection had positive tourniquet tests, and the test was also positive in those with DF (38%) almost as frequently as in those with DHF (45%). In the new WHO guidelines (2009), a positive tourniquet test is one of the criteria for diagnosis of dengue with or without warning signs (WHO 2009).

Among Vietnamese children, 5.6% did not have dengue but had positive tourniquet tests (Phuong *et al.* 2002). In this study, 16% of patients who did not have serological evidence of dengue infection had a positive tourniquet test. Therefore, without confirmation by serological tests, a significant minority of patients, whose true diagnoses are non-dengue infections, may be misdiagnosed as dengue and thus will miss the chance of specific treatment. Rickettsial infections, which are treatable with inexpensive doxycycline, were identified as the main cause of fever (approximately 27%) among Lao adults with negative blood cultures admitted to hospital in Vientiane (Phongmany *et al.* 2006). In this study, of 64 patients without serological evidence of dengue, 40 (62.5%) had typhus. Therefore, 17% of all Lao adult patients admitted with clinically suspected dengue had rickettsial disease, and without laboratory facilities, these patients may be misdiagnosed and not prescribed doxycycline.

A systematic review of clinical and laboratory features that distinguish dengue from other febrile illnesses suggested that patients with dengue had significantly lower platelet, white blood cell and neutrophil counts and a higher frequency of petechiae than patients with a non-dengue aetiology. Higher frequencies of myalgia, rash, haemorrhagic signs, lethargy/prostration and arthralgia and higher haematocrit were reported in adult patients with dengue (Potts & Rothman 2008). In this study, although admission bleeding signs and rash occurred significantly more frequently and platelet and white cell counts were significantly lower in those with dengue compared to those without, they were not specific indicators for dengue infection.

In conclusion, the conventional admission dengue tourniquet test adds relatively little value for the diagnosis of dengue among Lao adult inpatients with suspected dengue. Without serological diagnostic tests, it is very difficult to differentiate between dengue and other common sympatric infections such as scrub typhus, murine typhus, typhoid and Japanese encephalitis virus. However, a positive tourniquet test suggests that treatment of these alternative diagnoses may not be needed and that dengue fluid balance management and monitoring should be instituted – but the caveat of low sensitivity is crucial in that a negative tourniquet test does not exclude dengue. Until simple, rapid, accurate and inexpensive rapid diagnostic tests for diagnosis of these common infections are available, it will remain difficult for clinicians to correctly distinguish these common infections and provide appropriate treatment.
